# Supplementary material for: Pathophysiological Mechanisms by which Heat Stress Potentially Induces Kidney Inflammation and Chronic Kidney Disease in Sugarcane Workers
Source: Nutrients. 2020 Jun 2;12(6):1639. doi: 10.3390/nu12061639 (PMC7352879; doi:10.3390/nu12061639)
Supplement: Supplementary file 1 [file nutrients-12-01639-s001.pdf]

nutrients

**Supplement Table S1: Background data for Ingenio El Angel, El Salvador.**

Participation data for Ingenio El Angel, El Salvador cohort Year 2 (2015-2016).

| | | Number of Cutters |
|---|---|---|
| **Baseline measurements** | | 334 |
| Number of serum creatinine measurements during harvest year 2, (N) | 0 | 75 |
| | 1 | 28 |
| | 2 | 79 |
| | 3 | 152 |

Descriptive data concerning Ingenio El Angel, El Salvador cohort Year 2 (2015-2016) - Subjects with ≥1 measurements during harvest.

| N | 259 |
|---|---|
| Sex, N women (%) | 46 (18%) |
| Age (years), median (IQR) | 32 (24–43) |
| Baseline eGFR (ml/min/1.73 $m^2$), median (IQR) | 90 (67–108) |

## Supplement Table S2: Results from women in El Salvador, year 2

Kidney injury and risk factors among female sugarcane cutters.

| | **El Salvador** | |
|---|---|---|
| | **Worker follow-up occasions** | |
| | **Total** | **IKI** |
| | Age | |
| 18–30 | 42 | 0 |
| 31–40 | 49 | 2 (4%) |
| 41–50 | 12 | 0 |
| >50 | 17 | 5 (29%) |
| | Baseline eGFR | |
| >90 | 54 | 0 |
| 90–60 | 59 | 5 (8%) |
| 45–60 | 7 | 2 (29%) |
| <45 | 0 | - |
| | Baseline CRP (mg/L) | |
| <3 | 77 | 3 (4%) |
| 3–10 | 38 | 4 (11%) |
| 10–20 | 5 | 0 |
| >20 | 0 | - |
| | Follow-up CRP (mg/L) | |
| <3 | 62 | 1 (2%) |
| 3–10 | 48 | 4 (8%) |
| 10–20 | 6 | 0 |
| >20 | 4 | 2 (50%) |
| | Baseline Mg (mmol/L) | |
| ≥0.7 | 112 | 5 (4%) |
| <0.7 | 8 | 2 (25%) |
| | Baseline K | |
| ≥3.5 | 118 | 7 (6%) |
| <3.5 | 2 | 0 |
| | Follow-up K | |
| ≥3.5 | 109 | 5 (5%) |
| <3.5 | 11 | 2 (2%) |

Kidney injury and continuous biochemical parameters in female sugarcane cutters.

| | | Median (IQR) | | |
|---|---|---|---|---|
| | | **Baseline** | **Non IKI Day** | **IKI Day** |
| Worker-days | N | 46 | 113 | 7 |
| CRP | mg/dL | 1.6 (0.9–4.0) | 2.8 (1.5–5.7) | 6.0 (3.8–28) |
| Uric acid | mmol/L | 279 (215–332) | 265 (219–308) | 370 (388–434) |
| CPK | µkat/L | 1.6 (1.2–1.9) | 2.2 (1.8–2.8) | 3.1 (2.1–5.3) |

**Supplement Table S3: Multivariate regression analyses**

| Nicaragua (Adelante Cohort) | | | | | | |
|---|---|---|---|---|---|---|
| Worker-harvests | | | | | | |
| | **Total** | **IKI** | **Incidence Ratio (IR) (95% CI)** | **IR Adjusted for Job and Year (95% CI)** | **IR Adjusted NSAID, CRP and Fever (95% CI)** | **IR Adjusted for Sugary Drink, Water and Boli Intake (95% CI)** |
| Age (years) | | | | | | |
| 18-30 | 320 | 32 (10%) | ref | Ref | | |
| 31–40 | 155 | 16 (10%) | 1.0 (0.6–1.9) | 0.9 (0.5–1.7) | | |
| 41–50 | 43 | 5 (12%) | 1.2 (0.5–3.0) | 1.0 (0.4–2.7) | | |
| >50 | 14 | 0 (0%) | NA | NA | | |
| eGFR, baseline (mL/min/1.73m$^2$) | | | | | | |
| >90 | 358 | 29 (8%) | Ref | Ref | | |
| 90–60 | 163 | 23 (14%) | 1.7 (1.0–3.0) | 1.5 (0.9–2.6) | | |
| 45–60 | 10 | 1 (10%) | 1.2 (0.2–9.1) | 1.0 (0.1–7.7) | | |
| <45 | 1 | 0 (0%) | NA | NA | | |
| CRP, baseline (mg/L) | | | | | | |
| <3 | 423 | 40 (9%) | Ref | Ref | | |
| 3–10 | 85 | 12 (14%) | 1.5 (0.8–2.8) | 1.5 (0.8–2.8) | | |
| 10–20 | 12 | 0 (0%) | NA | NA | | |
| >20 | 12 | 1 (8%) | 0.9 (0.1–6.4) | 1.1 (0.1–7.9) | | |
| CRP, follow-up (mg/L) | | | | | | |
| <3 | 340 | 10 (3%) | Ref | Ref | Ref | |
| 3–10 | 142 | 14 (10%) | 3.4 (1.5–7.5) | 2.8 (1.2–6.4) | 3.5 (1.5–8.2) | |
| 10–20 | 24 | 9 (38%) | 13 (5–31) | 11 (4.5–28) | 12 (5–31) | |
| >20 | 26 | 20 (77%) | 26 (12–56) | 21 (10–45) | 26 (12–60) | |
| NSAID use at least once per week† | | | | | | |
| No | 444 | 37 (8%) | Ref | Ref | Ref | |
| Yes | 88 | 16 (18%) | 2.2 (1.2–3.9) | 1.8 (1.0–3.3) | 2.0 (1.1–3.7) | |
| Incident fever in past week(s) †† | | | | | | |
| No | 463 | 40 (9%) | Ref | Ref | Ref | |
| Yes | 43 | 11 (26%) | 3.0 (1.5–5.8) | 2.7 (1.4–5.3) | 1.1 (0.5–2.2) | |

| | | | | | |
|---|---|---|---|---|---|
| Sugary drink intake (L) | | | | | |
| <0.2 | 64 | 2 (3%) | Ref | Ref | Ref |
| 0.2–1 | 312 | 30 (10%) | 3.1 (0.7–13) | 3.3 (0.8–14) | 3.0 (0.7–12) |
| >1 | 155 | 21 (14%) | 4.3 (1.0–18) | 4.6 (1.1–20) | 4.0 (0.9–17) |
| Morning boli intake (N of 300 mL electrolyte sachets) | | | | | |
| 0 | 125 | 18 (14%) | Ref | Ref | Ref |
| 0–3 | 214 | 23 (11%) | 0.7 (0.4–1.4) | 0.8 (0.4–1.4) | 0.8 (0.4–1.6) |
| ≥3 | 180 | 12 (7%) | 0.4 (0.2–0.9) | 0.5 (0.2–1.1) | 0.5 (0.2–1.1) |
| Morning water intake (L) | | | | | |
| 0–2 | 76 | 12 (16%) | Ref | Ref | Ref |
| 2–5 | 209 | 18 (9%) | 0.5 (0.3–1.1) | 0.6 (0.3–1.2) | 0.7 (0.3–1.5) |
| >5 | 246 | 23 (9%) | 0.6 (0.3–1.2) | 0.8 (0.4–1.7) | 0.9 (0.4–2.0) |

Job: seed cutter and burned cane cutter. Year: 2017–2018 and 2018–2019. NSAID: Non-steroidal anti-inflammatory drugs. CRP: C-reactive protein.
